# *Drosophila* photoreceptor systems converge in arousal neurons and confer light responsive robustness

**DOI:** 10.3389/fnins.2023.1160353

**Published:** 2023-05-19

**Authors:** David D. Au, Jenny C. Liu, Soo Jee Park, Thanh H. Nguyen, Mia Dimalanta, Alexander J. Foden, Todd C. Holmes

**Affiliations:** ^1^Department of Physiology and Biophysics, School of Medicine, University of California, Irvine, Irvine, CA, United States; ^2^Center for Neural Circuit Mapping, School of Medicine, University of California, Irvine, Irvine, CA, United States

**Keywords:** cryptochrome, external rhodopsin, internal rhodopsin-7, non-image forming vision, electrophysiology, light arousal behavior, *Drosophila melanogaster*, photoreceptor circuit

## Abstract

Lateral ventral neurons (LNvs) in the fly circadian neural circuit mediate behaviors other than clock resetting, including light-activated acute arousal. Converging sensory inputs often confer functional redundancy. The LNvs have three distinct light input pathways: (1) cell autonomously expressed cryptochrome (CRY), (2) rhodopsin 7 (Rh7), and (3) synaptic inputs from the eyes and other external photoreceptors that express opsins and CRY. We explored the relative photoelectrical and behavioral input contributions of these three photoreceptor systems to determine their functional impact in flies. Patch-clamp electrophysiology measuring light evoked firing frequency (FF) was performed on large LNvs (l-LNvs) in response to UV (365 nm), violet (405 nm), blue (450 nm), or red (635 nm) LED light stimulation, testing controls versus mutants that lack photoreceptor inputs *gl60j*, *cry-null*, *rh7-null*, and double mutant *gl60j-cry-null* flies. For UV, violet, and blue short wavelength light inputs, all photoreceptor mutants show significantly attenuated action potential FF responses measured in the l-LNv. In contrast, red light FF responses are only significantly attenuated in double mutant *gl60j-cry-null* flies. We used a light-pulse arousal assay to compare behavioral responses to UV, violet, blue and red light of control and light input mutants, measuring the awakening arousal response of flies during subjective nighttime at two different intensities to capture potential threshold differences (10 and 400 μW/cm^2^). The light arousal behavioral results are similar to the electrophysiological results, showing significant attenuation of behavioral light responses for mutants compared to control. These results show that the different LNv convergent photoreceptor systems are integrated and together confer functional redundancy for light evoked behavioral arousal.

## 1. Introduction

Light provides sensory cues to many animals for navigating their environment. In insects like *Drosophila*, short-wavelength light has robust effects on visual behaviors, such as circadian entrainment, phototaxis, sleep/wake, and arousal that are mediated in part by non-image forming mechanisms based on two deep-brain photopigments: cryptochrome (CRY) and rhodopsin 7 (Rh7) (Stanewsky et al., 1998; Emery et al., 2000; Sheeba et al., 2008a; Kumar et al., 2012; Fogle et al., 2015; Baik et al., 2017, 2018, 2019b; Kistenpfennig et al., 2017; Ni et al., 2017; Senthilan et al., 2019). CRY is a light-sensitive photopigment that was initially identified in flies based on its role in light entraining fly circadian rhythm. CRY binds to TIMELESS (TIM) and PERIOD (PER) clock protein heteromultimeric complexes. Light-activated conformational changes of CRY’s C-terminal tail initiates a degradation cascade of co-complexed clock protein TIM and PER, thus calibrating/resetting the transcription-translation loop circadian clock (Ceriani et al., 1999; Koh et al., 2006; Peschel et al., 2009). CRY photoactivation also depends on electron transfer between multiple tryptophan residues embedded within the structure that result in photoreduction of CRY’s chromophore, flavin adenine dinucleotide (FAD) (Hoang et al., 2008; Zoltowski et al., 2011; Czarna et al., 2013; Levy et al., 2013; Vaidya et al., 2013; Lin et al., 2018; Baik et al., 2019a). CRY photoactivation also evokes robust increases in neuronal electrical excitability via redox coupled interactions with voltage-gated potassium channel beta subunits (Kvβ) hyperkinetic (Hk) (Fogle et al., 2011, 2015; Baik et al., 2017). Rh7 is a spectrally broad bistable photopigment with an absorbance peak around violet (∼400 nm) light and functions via a Gq/PLC (Gq class of G-proteins that couple to phospholipase C) phototransduction pathway (Ni et al., 2017; Sakai et al., 2017). While measurable using spectroscopic biophysical methods, functional Rh7 bistability is not fully explored at present. Both CRY and Rh7 are highly expressed in many of the neurons of the circadian/arousal neural circuit (Emery et al., 2000; Klarsfeld, 2004; Benito et al., 2008; Sheeba et al., 2008a; Yoshii et al., 2008; Fogle et al., 2011; Ni et al., 2017), including the lateral ventral neurons (LNvs), which use light input to tune many physiological and behavioral processes of the fly as noted above (Parisky et al., 2008; Shang et al., 2008; Sheeba et al., 2008a,^2010^; Liu et al., 2014; Fogle et al., 2015; Muraro and Ceriani, 2015; Buhl et al., 2016; Potdar and Sheeba, 2018; Chaturvedi et al., 2022). Flies and other insects also navigate their environments using six external rhodopsin photoreceptors found in the compound eyes, the Hofbauer-Buchner (HB) eyelet, and the ocelli that contribute to image-forming and non-image forming visual processes. Together, six rhodopsin photopigments, rhodopsin 1-6 (Rh1-6), mediate a broad range of spectral sensitivity from UV to red (∼300–630 nm) light (Kirschfeld et al., 1978, 1977; Feiler et al., 1992; Salcedo et al., 1999; Sharkey et al., 2020). CRY and Rh7 are also expressed in these external photoreceptor structures, where they may play a role in modulating visual sensitivity gain control (Senthilan et al., 2019). Senthilan et al. (2019) find evidence for Rh7 mRNA expression in brain neurons that is approximately equivalent to Rh7 mRNA expression in eyes, and immunocytochemical staining of Rh7 in the eyes but not in brain neurons. In contrast, Ni et al. (2017) shows robust Rh7 immunocytochemical staining in brain neurons including the LNv. Thus, for this study we were particularly interested in seeing whether Rh7 mediates light responses in the l-LNv and the responses in the absence of CRY and eye photoreceptors. Previous work shows that circadian photoreception and light attraction/avoidance behaviors are coordinately regulated by all three cell-autonomous photoreceptive pathways (CRY, Rh7, external photoreceptors), which input to the LNvs, and provide functional redundancy for these important behaviors (Emery et al., 1998; Stanewsky et al., 1998; Helfrich-Förster et al., 2001, 2002; Malpel et al., 2002; Rieger et al., 2003; Klarsfeld, 2004; Veleri et al., 2007; Sheeba et al., 2008a; Kistenpfennig et al., 2012; Schlichting et al., 2014, 2015, 2016; Saint-Charles et al., 2016; Ni et al., 2017; Baik et al., 2018, 2019b; Lazopulo et al., 2019).

Most of the LNv light-activated arousal neurons express the circadian neuropeptide pigment-dispersing factor (PDF) and can be further categorized as small and large (s-LNvs and l-LNvs, respectively) that are each uniquely capable of transmitting light information that contribute to endogenous circadian timekeeping or wakefulness/arousal. Light-activated s-LNvs trigger the release of PDF to entrain dorsal neurons (DNs), lateral dorsal neurons (LNds), and other circadian pacemaker neurons, while l-LNvs receive inputs from CRY, Rh7, or the external photoreceptors to trigger PDF release in the accessory medulla (aME) to signal light information to s-LNvs, LNds, and other clock neurons (Helfrich-Förster et al., 2007; Cusumano et al., 2009; Yoshii et al., 2009, 2016; Guo et al., 2014; Eck et al., 2016; Schlichting et al., 2016). Additionally, l-LNvs exhibit both rapid and long-lasting excitatory electrophysiological events upon short-wavelength light exposure as marked by an increase in firing frequency (FF) and membrane depolarization lasting tens of seconds from stimulus onset (Sheeba et al., 2008b; Fogle et al., 2011, 2015; Baik et al., 2017, 2019a). This phenomenon is thought to be driven primarily by the CRY/Hk and Rh7 photoreceptor systems. However, l-LNvs also exhibit acute responses to red light (∼635 nm) that, albeit weaker, persist in a *cry-null* system (Baik et al., 2019a; Au et al., 2022). In terms of l-LNv photoexcitability, this suggests a possible input contribution from red-sensitive rhodopsin photopigments (Rh1 and Rh6) from the external photoreceptor structures or direct effects mediated by CRY and/or Rh7. While circadian photoentrainment is primarily modulated by CRY, flies are still able to entrain to light:dark (LD) cycles in a CRY-independent manner, also suggesting that light inputs to LNvs and the rest of the clock circuitry is mediated by external opsin-based photoreceptor structures and/or Rh7 (Ni et al., 2017, but see also Kistenpfennig et al., 2017). Further evidence suggests that different properties of light (intensity, exposure timing, spectral composition) recruit different photoreceptors for photoentrainment (Chatterjee et al., 2018) and light attraction/avoidance behaviors (Baik et al., 2019b). Anatomically, external photoreceptor structures project either directly into the aME or indirectly via lamina monopolar cells that project to the aME (Behnia and Desplan, 2015). It has been proposed that the aME acts as a central hub for parallel light input circuits from the external photoreceptor system to the clock circuit for photoentrainment (Li et al., 2018), but the extent of how all three photoreceptor systems functionally contribute to light evoked neuronal photoexcitation and behavioral arousal remain largely unknown.

Here, we comprehensively explore the functional contributions of the light input pathways from the three distinct external and internal photoreceptor systems that activate LNvs electrical excitability and the relative contributions of these three distinct photoreceptor systems to the fly’s arousal responses to UV, violet, blue, and red light stimuli. We employ the light-evoked whole-cell current clamp electrophysiology assay to measure l-LNvs responses to intensity matched UV, violet, blue, and red light, comparing control versus fly mutants that lack photoreceptor inputs *gl60j*, *cry-null*, *rh7-null*, and double mutant *gl60j-cry-null* flies. In a parallel set of studies, we use a light-pulse arousal assay to measure behavioral responses to intensity matched UV, violet, blue, and red light, measuring light-triggered wakefulness from sleep for controls and each fly mutant at two different levels of light intensity. We find that all photoreceptor systems functionally integrate in l-LNvs to enable light-evoked electrophysiological excitability. We find similarly that all light input channels contribute to arousal behavioral responses to light. Identifying a functional connection between each of the fly photoreceptor systems strengthens an emerging model that insect image-forming and non-image forming visual processes work together in a coordinate fashion to mediate complex light-evoked behaviors.

## 2. Materials and methods

### 2.1. Experimental animals

Our experimental flies were allowed *ad libitum* access to a standard food media consisting of yeast, cornmeal, and agar at 25 ± 1°C and 40–60% relative humidity in 12:12 h light:dark cycles during behavioral experiments. All flies used in the experiments are 3 to 4-day post-eclosion adult male flies. We obtained our *rh7-null*, *gl60j*, and *gl60j-cry-null* mutant flies through a prior collaboration with Craig Montell of UC Santa Barbara, and *cry-null* mutant flies from Amita Seghal of University of Pennsylvania. Once obtained, we crossed each photoreceptor mutant with a *pdfGAL4-p12c* driver line to drive expression of green fluorescent protein (GFP) in all PDF+ neurons in order to visualize l-LNvs for patch-clamp electrophysiology. The *p12c* line is a stable expression line that we previously developed in our lab to mark LNvs using GFP expression driven by *pdfGAL4*. Large lateral ventral neurons were identified by size, morphology, and anatomical positioning. Our wild-type control is the *pdfGAL4-p12c* driver line.

### 2.2. Light-evoked neuronal electrophysiology

We adapted our light-evoked potential electrophysiology assays established in Baik et al. (2019a) to measure adult male fly brain’s large lateral ventral neuronal responses to various light stimuli. Our *ex vivo* brain preparations retain residual photoreceptor tissue from the compound eyes so as to avoid damaging the preparation and the input circuitry of the neurons we record from. This brain preparation closely follows the procedure outlined in Gu and O’Dowd (2006). Flies are dissected in an external recording solution comprised of the following components: 122 mM NaCl, 3 mM KCl, 1.8 mM CaCl_2_, 0.8 mM MgCl_2_, 5 mM glucose, 10 mM HEPES, and calibrated to a pH of 7.2 ± 0.02 and osmolarity of 250–255 mOsm. The internal recording solution is comprised of the following components: 102 mM Kgluconate, 17 mM NaCl, 0.085 mM CaCl_2_, 1.7 mM MgCl_2_ (hexahydrate), 8.5 mM HEPES, 0.94 mM EGTA, and is calibrated to a pH of 7.2 ± 0.02 and osmolarity of 232–235 mOsm. Our custom multichannel LED source (Prizmatix/Stanford Photonics, Palo Alto, CA, USA) is fitted onto an Olympus BX51 WI microscope and was used as our primary light source for light-evoked potential recordings. The LED are tuned to the following wavelengths of color: UV (365 nm), violet (405 nm), blue (450 nm), and red (635 nm), and all exposures were set to an intensity of 200 μW/cm^2^ by use of a Newport 842-PE Power/Energy meter. Transistor-transistor-logic (TTL) triggered LEDs programmed by the data acquisition software, pClamp (molecular dynamics), enabled rapid on/off light stimuli with the following protocol: 50 s of dark for baseline recording, 5 s of colored-light stimulation, then 95 s of inter-pulse darkness for FF and membrane potential baseline recovery. We measure five continuously repeated sweeps per recording to allow for greater statistical confidence in our measurements, which are analyzed as follows: FF is determined by counting spikes per 10 s interval per 100 s sweep, then calculated as a FF ratio by the average number of spikes during lights on over the average number of spikes per 10 s interval pre-light stimulus. These ratios are averaged across the five repeated sweeps per recording for all samples of the same light-stimulus in the genotype set. This custom light-evoked potential protocol allows greater measurements for kinetically robust light-evoked effects in our samples.

### 2.3. Light-pulse arousal behavioral assay

Standard LD light pulse arousal assays were conducted from previously established and adapted protocols (Baik et al., 2017). The locomotor activity of individual flies was measured using the TriKinetics Locomotor Activity Monitoring System via infrared beam-crossing, recording total crosses in 1-min bins. Percentage activity and statistics were measured using Microsoft Excel. Custom LED fixtures were built using Waveform Lighting blue, violet, UV, and red LEDs with a narrow peak wavelength of 450, 405, 365, and 635 nm, respectively, and intensity-tuned to 10 μW/cm^2^ and 400 μW/cm^2^ for low and high intensity light exposures, respectively.

### 2.4. Quantification and statistical analysis

All reported values are represented as mean ± SEM. Values of *n* refer to the total number of experimental flies tested over all replicates of an experiment (minimum of three replicates). Firing frequency values are calculated as a ratio of spikes during the 5 s of lights on/average baseline firing rate binned in 10 s increments. Statistical tests were performed using Minitab, Matlab, and Microsoft Excel software. Statistical analysis began with performing an Anderson–Darling normality tests to determine normality of data. Variance was determined using F-tests for normally distributed data, then significance was determined using two-sample, one-tailed *t*-tests with alpha values of 0.5 before pairwise correction. Significance for non-normal data was determined by Mann-Whitney U-tests. Spike firing quantifications were performed using custom Matlab scripts and Clampfit software. Multi-comparison tests leading to Type I error/false positives were mitigated by *p*-value adjustment based on false discovery rate (FDR, Benjamini and Hochberg, 1995). A standard FDR threshold of 0.1 was then implemented in order to indicate significance as an expected proportion of false positives that is no greater than 10%.

## 3. Results

### 3.1. Light excitation of arousal neurons to short-wavelength light relies on input coincident of multiple photoreceptor systems

Physiological and anatomical evidence indicates the convergence of multiple photoreceptor channels on the LNvs (Emery et al., 1998; Stanewsky et al., 1998; Helfrich-Förster et al., 2001, 2002; Malpel et al., 2002; Klarsfeld, 2004; Sheeba et al., 2008a; Schlichting et al., 2014, 2016; Behnia and Desplan, 2015; Ni et al., 2017; Chatterjee et al., 2018; Li et al., 2018; Baik et al., 2019b). CRY light activation is based successive redox reduction of its FAD starting at a base oxidized state with two absorption peaks corresponding to ultraviolet (UV, 365 nm) and blue (450 nm) light (Berndt et al., 2007; Bouly et al., 2007; Hoang et al., 2008). Higher reduction states of CRY confer light-evoked excitation by red light (635 nm) due to spectral absorption peak shifts. Light evoked redox transfer reactions mediated by FAD in CRY are transduced to changes in membrane potential and neuronal excitability through voltage gated potassium channel beta subunit, hyperkinetic (Sheeba et al., 2008b; Fogle et al., 2011, 2015; Baik et al., 2017). Rh7 exhibits a very broad absorption that peaks at violet light (405 nm) (Kistenpfennig et al., 2017; Ni et al., 2017). In order to test the relative contributions of different photoreceptor systems on l-LNv photoexcitability to short-wavelength light, we performed whole-cell current-clamp electrophysiology using 200 μW/cm^2^ of UV (365 nm), violet (405 nm), and blue (450 nm) LED light. To test the contribution of each photoreceptor system to circadian/arousal neuronal photoexcitability to these short wavelengths, we measured the electrophysiological light responses of l-LNvs as a ratio of action potential FF during lights on/lights off, comparing control wild-type *p12c* with recordings of genetic knockouts *cry-null*, *rh7-null*, total external photoreceptor knockout *gl60j*, and a double-mutant *gl60j-cry-null* photoreceptor mutant flies using the whole-cell patch-clamp electrophysiology configuration. Recordings were performed on l-LNvs from each photoreceptor knockout group with 50 s of dark, 5 s exposures of 200 μW/cm^2^ LED light for each wavelength of light, and 95 s of dark inter-pulse intervals in order to measure post-stimulus decay back to baseline. Each recording trace was repeated 5 times and FF ratios are reported as an average of all traces of all recordings for each group of parameters.

**FIGURE 1:**
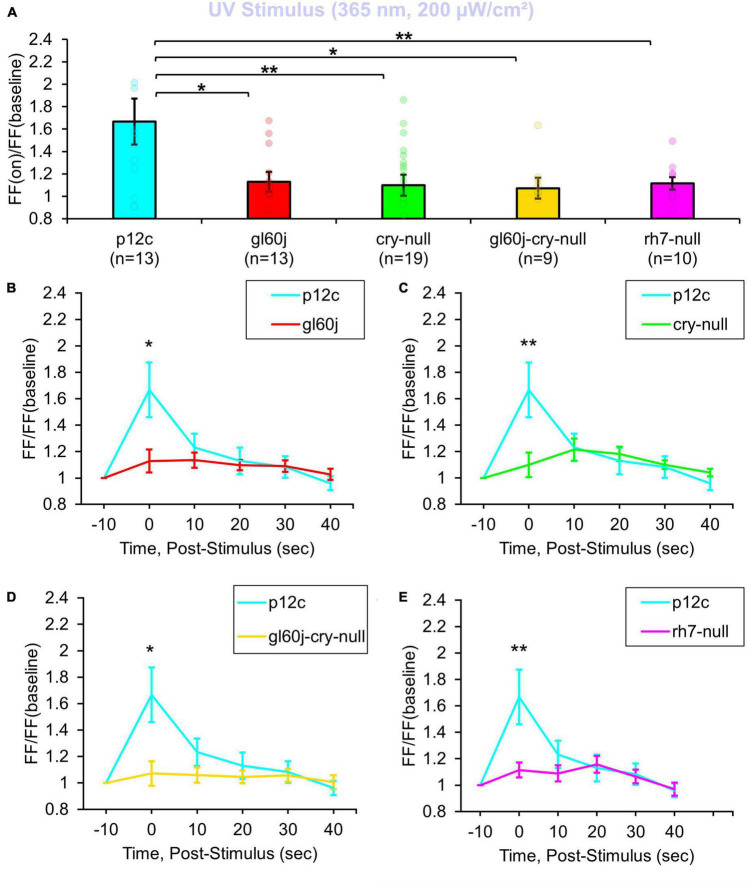
All photoreceptor mutants show an attenuated UV light firing frequency (FF) compared to native expressed *Drosophila* CRY. **(A)** Firing frequency response of *p12c* (blue column, *n* = 13) versus *gl60j* (red column, *n* = 13), *cry-null* (green column, *n* = 19), *gl60j-cry-null* (yellow column, *n* = 9), and *rh7-null* (violet column, *n* = 10) with 5 s UV (365 nm, 200 μW/cm^2^) light stimulus. Post-stimulus FF response in 10 min bins for **(B)** *p12c* (blue trace) vs. *gl60j* (red trace), **(C)** *p12c* vs. *cry-null* (green trace), **(D)** *p12c* vs. *gl60j-cry-null* (yellow trace), and **(E)** *p12c* vs. *rh7-null* (violet trace). Data are plotted as average ± SEM. Pairwise comparison was analyzed using two-sample *t*-test with FDR adjustment. *p < 0.1 and **p < 0.05.

A total of 200 μW/cm^2^ UV (365 nm) LED light evokes a robust 1.6-fold FF increase in recordings of *p12c* control l-LNvs (Figure 1A blue column). As expected, intensity matched UV light electrophysiological responses of l-LNvs are significantly attenuated in neurons recorded from *cry-null* fly brains (Figure 1A, blue column vs. green column) as 365 nm corresponds to one of the principal spectral absorption peaks for the base oxidized state of CRY. Intensity matched UV light electrophysiological responses of l-LNvs also are significantly attenuated to a similar degree in neurons recorded from *gl60j* and *rh7-null* fly brains (Figure 1A, blue column vs. red column and violet column, respectively). This indicates that opsin-based phototransduction in eyes/external photoreceptors and cell autonomously expressed Rh7 also contribute to the l-LNv electrophysiological responses to UV light. The UV photoresponse of the double mutant *gl60j-cry-null* is also significantly different from control *p12c* (Figure 1A, blue column vs. yellow column), in agreement with the attenuated UV light response trends measured from the single mutants *gl60j* and *cry-null*. Short term light exposure evokes long term subsequent increases in neuronal excitation in LNvs (Fogle et al., 2011, 2015; Baik et al., 2017, 2019a). In order to measure any lasting photoexcitatory effects post-stimulus, FF ratios for each knockout mutant are reported as 10 s intervals for 50 s post-stimulus: FF (10 s post-stimulus)/FF (baseline), FF (20 s post-stimulus)/FF (baseline), FF (30 s post-stimulus)/FF (baseline), FF (40 s post-stimulus)/FF (baseline), and FF (50 s post-stimulus)/FF (baseline). There are no significant increases in FF ratio post-stimulus when comparing *p12c* versus any of the knockout mutants (Figures 1B–E).

**FIGURE 2:**
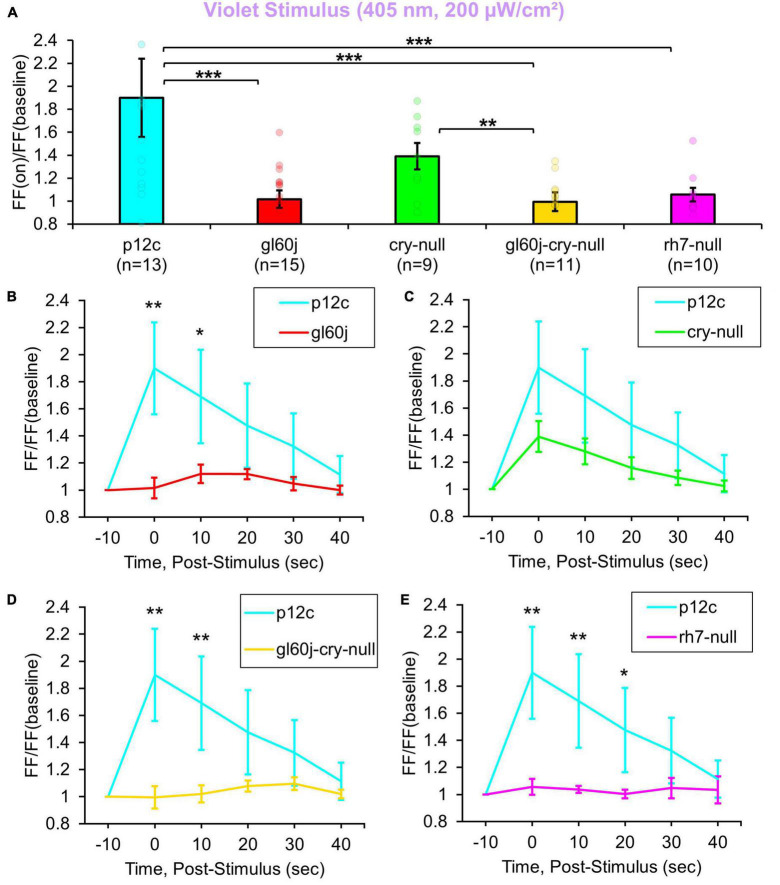
All photoreceptor mutants except *cry-null* show an attenuated violet light firing frequency (FF) compared to native expressed *Drosophila* CRY. **(A)** Firing frequency response of *p12c* (blue column, *n* = 13) versus *gl60j* (red column, *n* = 15), *cry-null* (green column, *n* = 9), *gl60j-cry-null* (yellow column, *n* = 11), and *rh7-null* (violet column, *n* = 10) with 5 s violet (405 nm, 200 μW/cm^2^) light stimulus. Post-stimulus FF response in 10 min bins for **(B)** *p12c* (blue trace) vs. *gl60j* (red trace), **(C)** *p12c* vs. *cry-null* (green trace), **(D)** *p12c* vs. *gl60j-cry-null* (yellow trace), and **(E)** *p12c* vs. *rh7-null* (violet trace). Data are plotted as average ± SEM. Pairwise comparison was analyzed using two-sample *t*-test with FDR adjustment. *p < 0.1, **p < 0.05, ***p < 0.01.

200 μW/cm^2^ of violet (405 nm) LED light evokes 1.8-fold increases in FF in control *p12c* l-LNvs (Figure 2A, blue column). In contrast, intensity matched l-LNv violet light responses in FF are significantly attenuated in l-LNv recordings of *gl60j*, *rh7-null*, and double mutant *gl60j-cry-null* neurons (Figure 2A, blue column vs. red column, violet column, and yellow column, respectively). Unsurprisingly, *cry-null* violet light responses in FF are not significantly different from control as there is an absorption trough at 405 nm for CRY, but *cry-null* violet light responses are significantly higher compared to the *gl60j-cry-null* response (Figure 2A, green column vs. yellow column, respectively). Comparing the *gl60j* versus the double mutant *gl60j-cry-null* recordings of violet evoked changes in FF, this result suggests the l-LNvs responsiveness to violet light depends entirely on external and cell autonomous opsin-based photoreceptors. In comparison to control *p12c*, the post-stimulus FF ratio for violet light for each genotype (Figures 2B–E) shows significant increases up to 10 s post-stimulus for *gl60j* and *gl60j-cry-null* responses (Figures 2B, D, respectively), and up to 20 s for *rh7-null* responses (Figure 2E), but no significant increases for cry-null responses (Figure 2C).

**FIGURE 3:**
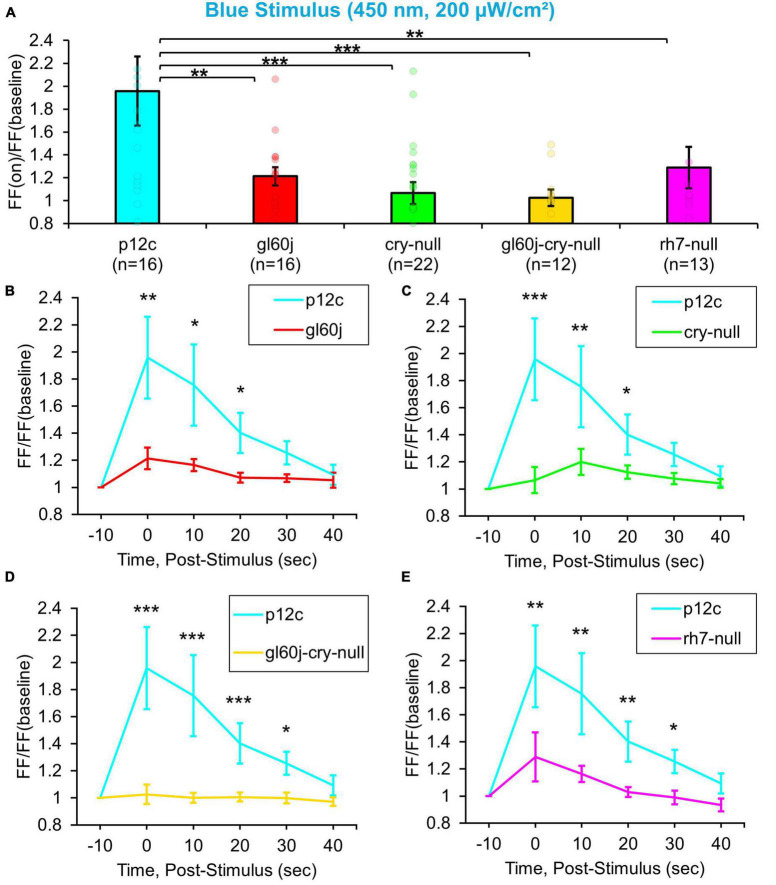
All photoreceptor mutants show an attenuated blue light firing frequency (FF) compared to native expressed *Drosophila* CRY. **(A)** Firing frequency response of *p12c* (blue column, *n* = 16) versus *gl60j* (red column, *n* = 16), *cry-null* (green column, *n* = 22), *gl60j-cry-null* (yellow column, *n* = 12), and *rh7-null* (violet column, *n* = 13) with 5 s blue (450 nm, 200 μW/cm^2^) light stimulus. Post-stimulus FF response in 10 min bins for **(B)** *p12c* (blue trace) vs. *gl60j* (red trace), **(C)** *p12c* vs. *cry-null* (green trace), **(D)** *p12c* vs. *gl60j-cry-null* (yellow trace), and **(E)** *p12c* vs. *rh7-null* (violet trace). Data are plotted as average ± SEM. Pairwise comparison was analyzed using two-sample *t*-test with FDR adjustment. *p < 0.1, **p < 0.05, ***p < 0.01.

Natively expressed CRY control *p12c* l-LNvs are robustly excited by blue light exposure, showing an almost a twofold increase in FF (Figure 3A, blue column), similar to results reported previously. Compared to *p12c* controls, the genetic absence of other photoreceptors/phototransducers including the *gl60j* mutation or *rh7-null* results in a significant attenuation of l-LNv responsiveness to blue light (Figure 3A, blue column vs. red column and violet column, respectively). Mutant *cry-null* show a significantly more attenuated response, as we have demonstrated previously (Figure 3A, blue column vs. green column). The double-mutant *gl60j-cry-null* exhibits the greatest attenuation of l-LNv responsiveness to blue light (Figure 3A, yellow column) suggesting a compounding effect from loss of photoreception from both systems. Blue light-responses are also long-lasting for the *p12c* control group compared to *gl60j* and *cry-null*, with *p12c* having a sustained FF ratio increase lasting up to 20 s post-stimulus (Figures 3B, C, respectively). In comparison to *rh7-null* and the double mutant *gl60j-cry-null*, the control blue light response persists for even longer, up to 30 s post-stimulus (Figures 3D, E, respectively).

### 3.2. External photoreceptors and cryptochrome dually contribute to mediate red light excitability in primary arousal neurons

**FIGURE 4:**
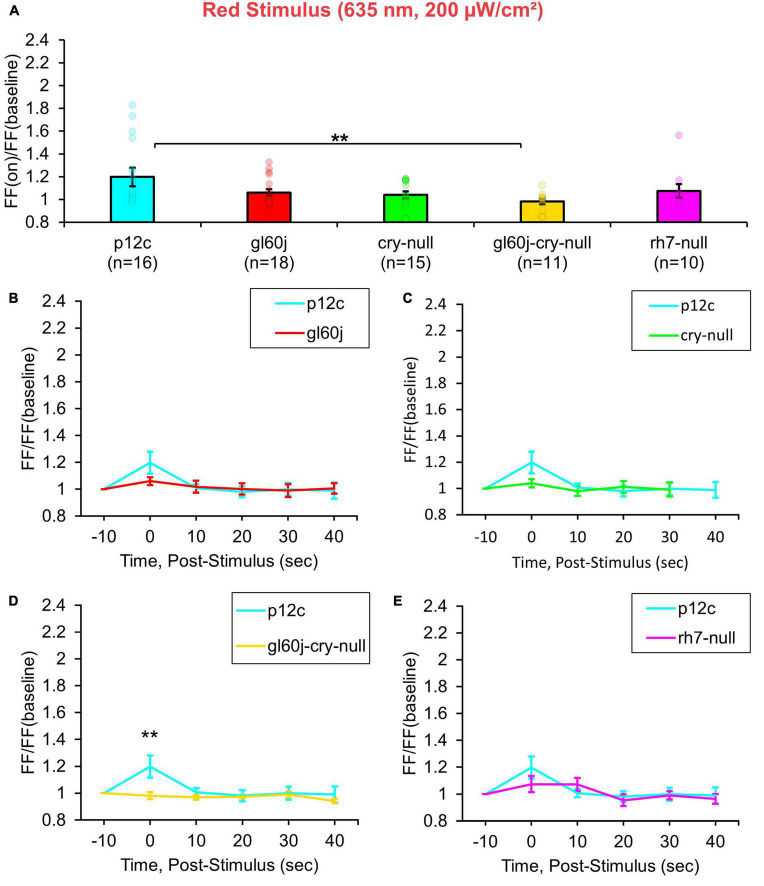
*gl60j-cry-null* photoreceptor mutants show an attenuated red light firing frequency (FF) compared to native expressed *Drosophila* CRY. **(A)** Firing frequency response of *p12c* (blue column, *n* = 16) versus *gl60j* (red column, *n* = 18), *cry-null* (green column, *n* = 15), *gl60j-cry-null* (yellow column, *n* = 11), and *rh7-null* (violet column, *n* = 10) with 5 s red (635 nm, 200 μW/cm^2^) light stimulus. Post-stimulus FF response in 10 min bins for **(B)** *p12c* (blue trace) vs. *gl60j* (red trace), **(C)** *p12c* vs. *cry-null* (green trace), **(D)** *p12c* vs. *gl60j-cry-null* (yellow trace), and **(E)** *p12c* vs. *rh7-null* (violet trace). Data are plotted as average ± SEM. Pairwise comparison was analyzed using two-sample *t*-test with FDR adjustment. **p < 0.05.

Multiple lines of evidence show that CRY also mediates acute red light responsiveness as measured by l-LNv electrophysiology (Baik et al., 2019a; Au et al., 2022). These surprising results suggest that CRY can be sufficiently reduced to reach long-wavelength light absorption *in vivo*. The only other known candidates for red light sensing in flies occur via red sensitive opsin-based photoreceptors expressed in the compound eyes including rhodopsin 1 and rhodopsin 6. Anatomical and physiological evidence suggest circumstantially that external photoreceptor systems directly input light information to the circadian/arousal neural circuits. Internally expressed Rh7 does contribute violet (405 nm) light sensing to l-LNvs, yet Rh7’s contribution to l-LNv input to other wavelengths of light is largely unexplored. A total of 200 μW/cm^2^ red light exposure evokes small but measurable acute increases in action potential FF in control *p12c* l-LNvs. In contrast, attenuated responses are measured in double knockout *gl60j-cry-null* l-LNvs (Figure 4A, blue column vs. yellow column). Red light evoked increases in FF quickly return to baseline firing within 10 s post-stimulus, indicating that l-LNv electrophysiological responses to red light are acute rather than long-lasting (Figure 4D, blue trace vs. yellow trace). Post-red stimulus plots for *gl60j*, *cry-null*, and *rh7-null* mutant groups show no significant differences in comparison to the *p12c* control (Figures 4B, C, E). A summary table of all genotypes FF responses (FF lights on/FF baseline no light) to light stimuli can be found on Table 1.

**TABLE 1:** Summary of firing frequency ratio for each genotype against each color of light stimulus.

	UV	Violet	Blue	Red
*p12c*	1.667	1.899	1.958	1.197
*gl60j*	1.129	1.016	1.213	1.06
*cry-null*	1.099	1.39	1.066	1.04
*gl60j-cry-null*	1.328	0.994	1.026	0.981
*rh7-null*	1.115	1.056	1.289	1.074

Firing frequency responses (FF lights on/FF baseline no light) of *p12c*, *gl60j*, *cry-null*, *gl60j-cry-null*, and *rh7-null* for UV, violet, blue, and red (200 μW/cm^2^ of 365, 405, 450, and 635 nm, respectively) light stimulus. The green shading represents larger FF light responses, while the white shading represents smaller FF light responses.

### 3.3. Light responses recorded from l-LNvs show no apparent time-of-day differences

**FIGURE 5:**
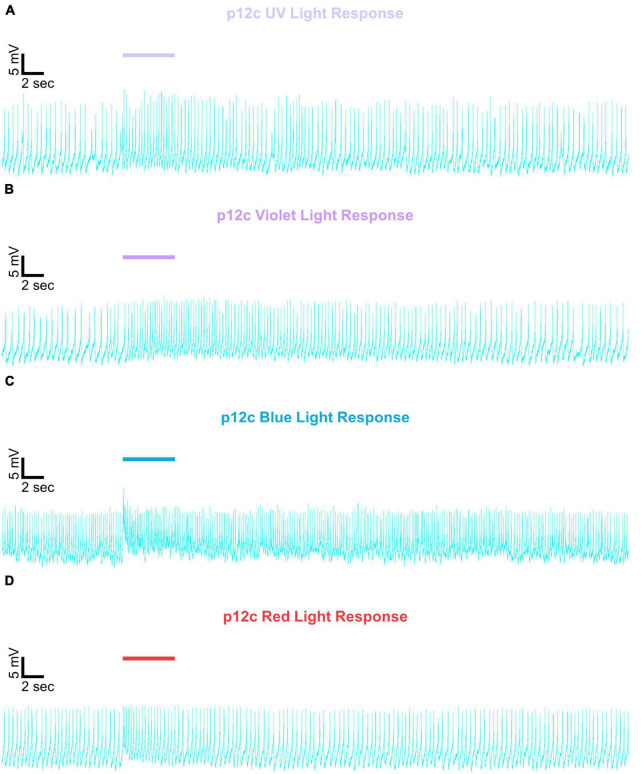
Representative voltage traces of l-LNvs electrophysiological responses to UV, violet, blue, and red light stimulus for native expressed *Drosophila* CRY. Representative voltage traces of the last 60 s of a patch-clamp recording of l-LNvs subjected to 5 s of **(A)** UV, **(B)** violet, **(C)** blue, and **(D)** red light stimulus for natively expressed *Drosophila* CRY, *p12c* (blue traces). Colored bars indicate 5 s of 200 μW/cm^2^ light stimulus. Vertical scale bars represent 5 mV and horizontal scale bars represent 2 s.

**FIGURE 6:**
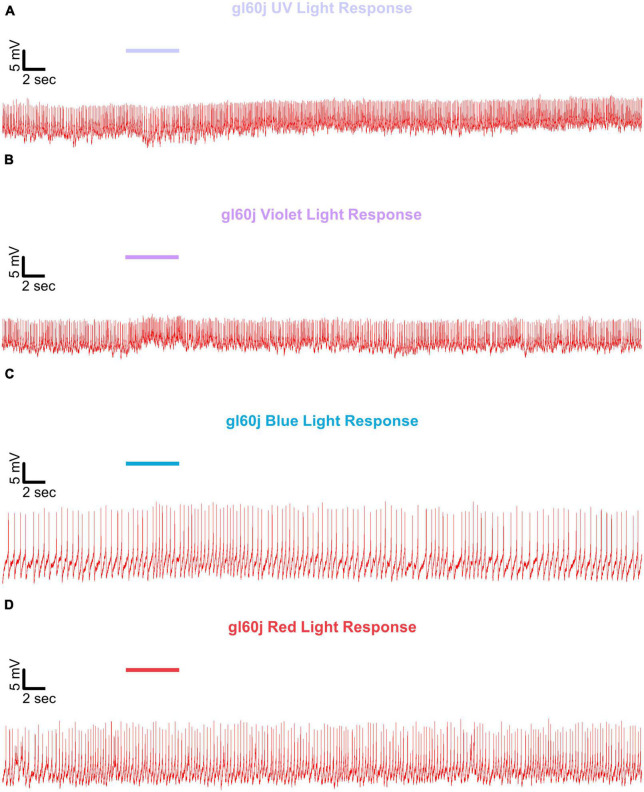
Representative voltage traces of l-LNvs electrophysiological responses to UV, violet, blue, and red light stimulus for *gl60j*. Representative voltage traces of the last 60 s of a patch-clamp recording of l-LNvs subjected to 5 s of **(A)** UV, **(B)** violet, **(C)** blue, and **(D)** red light stimulus for *gl60j* flies (red traces). Colored bars indicate 5 s of 200 μW/cm^2^ light stimulus. Vertical scale bars represent 5 mV and horizontal scale bars represent 2 s.

**FIGURE 7:**
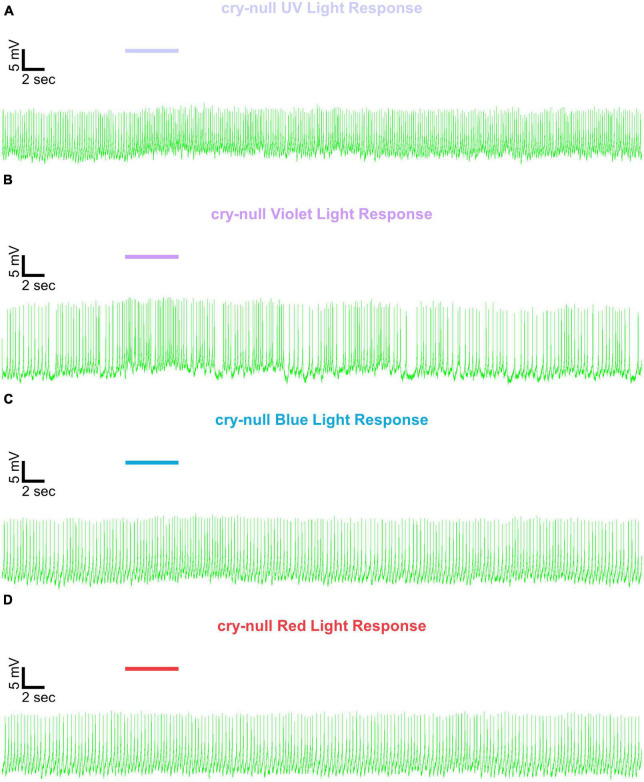
Representative voltage traces of l-LNvs electrophysiological responses to UV, violet, blue, and red light stimulus for *cry-null*. Representative voltage traces of the last 60 s of a patch-clamp recording of l-LNvs subjected to 5 s of **(A)** UV, **(B)** violet, **(C)** blue, and **(D)** red light stimulus for *cry-null* flies (green traces). Colored bars indicate 5 s of 200 μW/cm^2^ light stimulus. Vertical scale bars represent 5 mV and horizontal scale bars represent 2 s.

**FIGURE 8:**
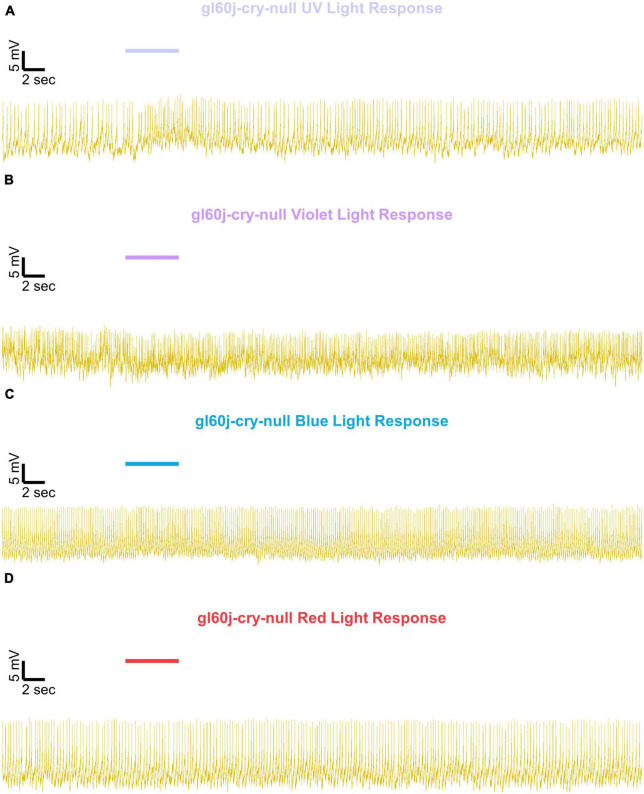
Representative voltage traces of l-LNvs electrophysiological responses to UV, violet, blue, and red light stimulus for *gl60j-cry-null*. Representative voltage traces of the last 60 s of a patch-clamp recording of l-LNvs subjected to 5 s of **(A)** UV, **(B)** violet, **(C)** blue, and **(D)** red light stimulus for *gl60j-cry-null* flies (yellow traces). Colored bars indicate 5 s of 200 μW/cm^2^ light stimulus. Vertical scale bars represent 5 mV and horizontal scale bars represent 2 s.

**FIGURE 9:**
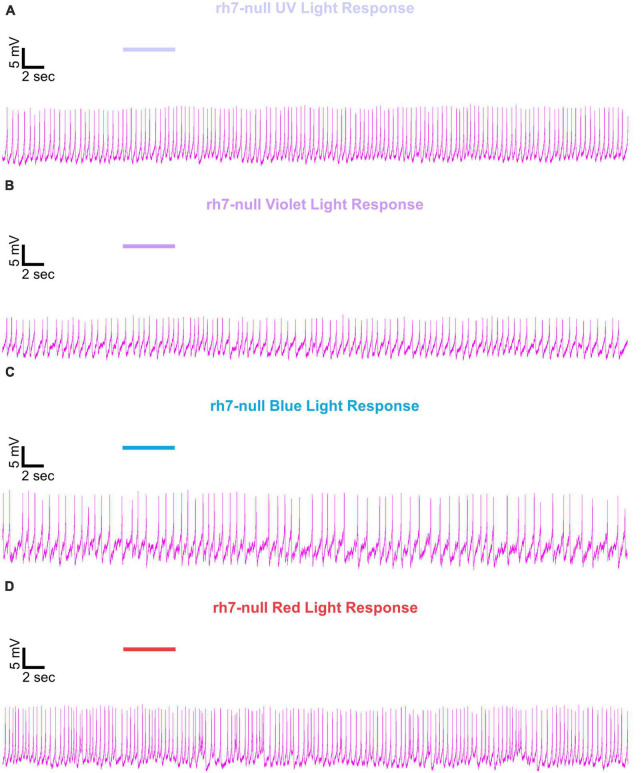
Representative voltage traces of l-LNvs electrophysiological responses to UV, violet, blue, and red light stimulus for *rh7-null*. Representative voltage traces of the last 60 s of a patch-clamp recording of l-LNvs subjected to 5 s of **(A)** UV, **(B)** violet, **(C)** blue, and **(D)** red light stimulus for *rh7-null* flies (violet traces). Colored bars indicate 5 s of 200 μW/cm^2^ light stimulus. Vertical scale bars represent 5 mV and horizontal scale bars represent 2 s.

Representative voltage traces with a 5 s baseline in dark, during the 5 s of 200 μW/cm^2^ red, blue, violet, or UV light stimulation, and 50 s post-stimuli show the increase in FF and duration of sustained excitation for *p12c* (Figure 5, blue traces), *gl60j* (Figure 6, red traces), *cry-null* (Figure 7, green traces), *gl60j-cry-null* (Figure 8, yellow traces), and *rh7-null* (Figure 9, violet traces). The colored bars indicate onset of 5 s of 200 μW/cm^2^ lights on and off during each recording. Most of the individual l-LNv recordings reveal predominately tonic action potential firing pattern, consistent with most other reports (Holmes et al., 2007; Cao and Nitabach, 2008; Sheeba et al., 2008b, 2010; McCarthy et al., 2011; Seluzicki et al., 2014; Flourakis and Allada, 2015; Flourakis et al., 2015; Fogle et al., 2015; Buhl et al., 2016, 2019; Baik et al., 2017, 2019a; Li et al., 2018; Smith et al., 2019; Au et al., 2022).

**FIGURE 10:**
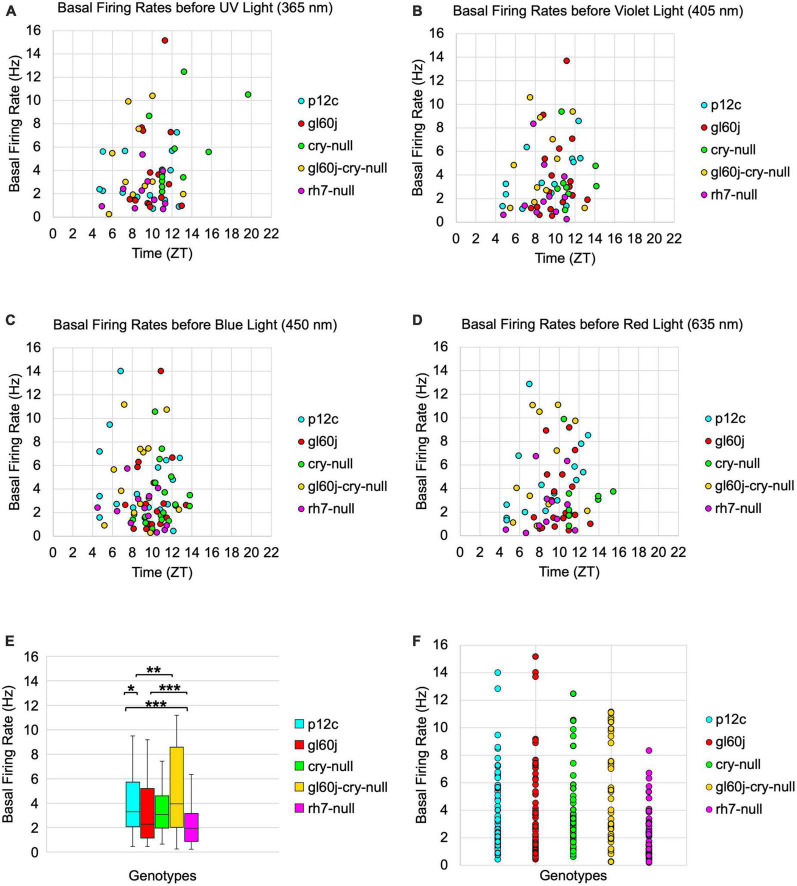
Basal firing rates are not equivalent across groups and there is no time-of-day dependent effect. Average basal firing rates of *p12c* (blue), *gl60j* (red), *cry-null* (green), *gl60j-cry-null* (yellow), and *rh7-null* (violet) before **(A)** UV, **(B)** violet, **(C)** blue, and **(D)** red light stimulus plotted against the relative time-of-day of each recording. **(E)** Box-and-whisker plot summary of the average basal firing rate for *p12c* [(*n* = 35) total, *n* (ZT0-12) = 30; *n* (ZT12-16) = 5], *gl60j* [(*n* = 22) total, *n* (ZT0-12) = 20; *n* (ZT12-16) = 2], *cry-null* [(*n* = 26) total, *n* (ZT0-12) = 14; *n* (ZT12-16) = 12], *gl60j-cry-null* [(*n* = 30) total, *n* (ZT0-12) = 22; *n* (ZT12-16) = 8], and *rh7-null* [(*n* = 30) total, *n* (ZT0-12) = 22; *n* (ZT12-16) = 8]. Median values are denoted by a solid black line within each box of the plot. **(F)** Individual data points for each genotype for all time points, showing the distribution of the data. Black *indicates FDR adjusted two-sample *t*-test *p* ≤ 0.01 vs. *p12c*. Data are represented as a range of means in a sample set ± maximum and minimum values within the set. One significance symbol, *p* ≤ 0.1; two significance symbols, *p* ≤ 0.05; three significance symbols, *p* ≤ 0.01.

To determine if the absence of CRY or the internal and external rhodopsin photoreceptors alters the basal FF of l-LNvs, we plotted basal firing rates across the time of day of the recordings (Figure 10). Scatter plots for the *p12c* control and all photoreceptor mutants pre-exposed to UV, violet, blue, or red light (Figures 10A–D, respectively) show no discernable correlation to time-of-day of the recording and FF baseline. However, average FF baseline for each group plotted as a box and whisker plot shows significantly lower baseline FF for *gl60j* and *rh7-null* mutants compared to the control *p12c* (Figure 10E, approximate average of 4.2 Hz for *p12c*, blue box vs. 3.6 Hz for *gl60j*, red box and 2.4 Hz for *rh7-null*, violet box). The double knockout *gl60j-cry-null* had a significantly higher FF baseline than *gl60j* (Figure 10E, approximate average of 5.2 Hz for *gl60j-cry-null*, yellow box vs. red box) but recordings from this genotype tend to be more unstable as indicated by the wide range of measured FF. Similarly, *cry-null* baseline FF is significantly higher than *rh7-null* baseline FF (Figure 10E, approximate average of 3.9 Hz for *cry-null*, green box vs. violet box). To provide a clear of the data distribution, individually plotted points for each genotype can be found on Figure 10F. These results show that removal of any opsin-based photoreceptor system results in a decrease in l-LNv baseline FF in absence of light, thus opsin-based photoreceptors modulate baseline circadian/arousal neuronal firing.

### 3.4. Photoreceptor mutant fly light-evoked arousal responses during sleep are significantly attenuated but not abolished, similar to the l-LNvs photoexcitatory defects

Non-imaging forming vision in flies is primarily mediated by CRY and Rh7 photoreceptors expressed in the LNv subset of circadian/arousal neurons are responsible for acute light-mediated behaviors such as arousal as well as circadian entrainment. Fly light evoked arousal responses occur over a range of wavelengths from 365–635 nm and vary with intensity. Though primarily short-wavelength photodetectors, CRY and Rh7 may also exhibit sensitivities reaching longer wavelength orange-red colored light. External photoreceptors in the compound eyes, ocelli, and Hofbauer-Buchner eyelet of flies express a wide range of opsin-based photoreceptors that further equip the fly with image-forming visual photoreception spanning the UV and visible light spectrum. Visual neural circuits downstream of external photoreceptors appear to synapse in the accessory medulla in the fly brain in close proximity to LNv circadian/arousal neurons and appear to integrate with CRY and Rh7 non-image forming visual mechanisms to modulate circadian entrainment to light and phototaxis/photoavoidance (Klarsfeld, 2004; Veleri et al., 2007; Helfrich-Förster, 2020). Whether these external photoreceptors contribute excitatory light color-specific information to mediate behavioral arousal responses remains unclear. We measured the acute behavioral responses of *p12c* control flies and photoreceptor mutant flies (*gl60j*, *cry-null*, double mutant *gl60j-cry-null*, and *rh7-null*) to three 5-min pulses of low (10 μW/cm^2^) or high (400 μW/cm^2^) red (635 nm), blue (450 nm), violet (405 nm), or UV (365 nm) LED light during subjective nighttime at time points ZT18, ZT19, and ZT20 for three consecutive nights. Scatter plots show the average% of flies that awaken across the 3 days of experiment per each light-pulse with responses from the first, second, and third pulses spanning each cluster from left, middle, and right, respectively. Average fly arousal responses do not significantly differ between consecutive nights of experiment.

**FIGURE 11:**
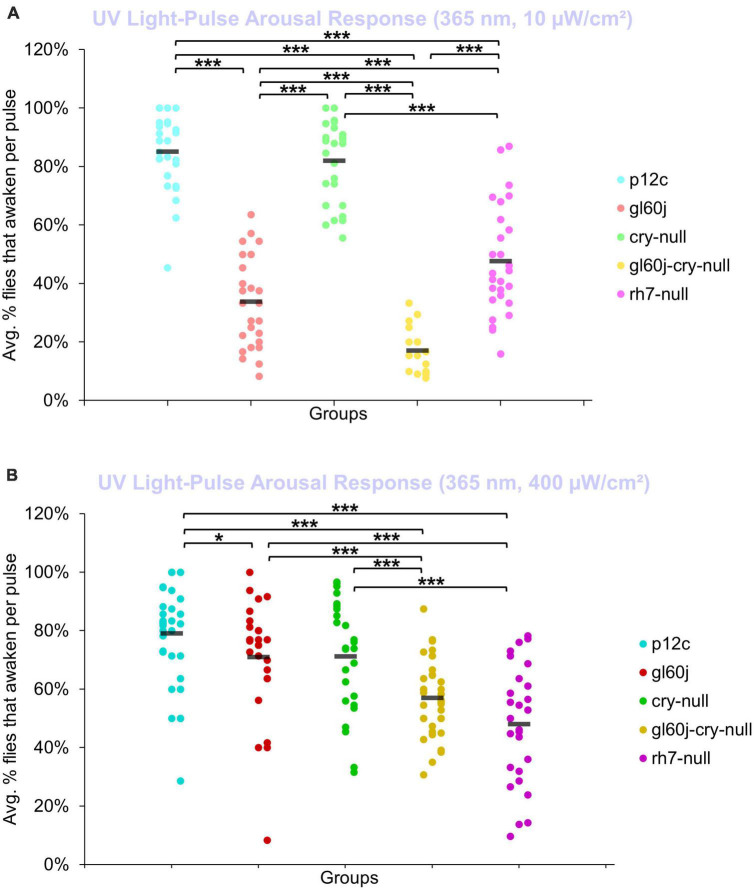
Low and high intensity UV light pulse arousal behavior is significantly attenuated in all photoreceptor mutants except *cry-null* compared to the control *p12c.* Three 5 min pulses of UV light were applied to flies during subjective nighttime (ZT18, ZT19, ZT20) for 3 days after 12:12 h LD entrainment to measure the arousal response of *p12c* (blue), *gl60j* (red), *cry-null* (green), *gl60j-cry-null* (yellow), and *rh7-null* (violet) flies for **(A)** low (10 μW/cm^2^) and **(B)** high (400 μW/cm^2^) light intensity. Scatter plots are grouped by average% of flies that awaken across the 3 days of light-pulse arousal experiment and separated as pulse 1 (left points for each group), pulse 2 (middle points for each group), and pulse 3 (right points for each group). Black bars indicate total average% flies that awaken across the 3 days and three pulses of light. Pairwise comparison was analyzed using two-sample *t*-test with FDR adjustment. *p < 0.1 and ***p < 0.01.

An average of nearly 85% of control *p12c* flies are aroused from sleep in response to low (10 μW/cm^2^) intensity UV light (Figure 11A, light blue points). All photoreceptor mutants except *cry-null* exhibit significantly lower arousal responses to low intensity UV light pulses relative to *p12c* controls (Figure 11A), suggesting that the light intensity threshold for CRY activation is higher than that for opsins. The double mutant *gl60j-cry-null* shows the greatest response attenuation of light evoked arousal to low intensity UV light pulses compared to all other genotypes (Figure 11A, light yellow points). Notably, *gl60j-cry-nulls* do not show significantly attenuated response in l-LNv photoexcitability to UV light at 20 fold higher light stimulus intensity (Figures 1A, D), suggesting possible additional UV sensing mechanisms for fly arousal. All photoreceptor mutants except *cry-null* exhibit a loss of arousal sensitivity compared to *p12c* in response to higher intensity UV light pulses (Figure 11B). However, both *gl60j* and *gl60j-cry-null* flies show significantly attenuated higher intensity UV light arousal responses (Figure 11B, dark red points and dark yellow points, respectively) and double mutant *gl60j-cry-null* flies show significantly attenuated higher intensity UV light arousal responses relative to *gl60j* alone, indicating that CRY does contribute to UV light evoked arousal. Mutant *rh7-null* flies show the greatest degree of attenuation of higher intensity UV light arousal responses (Figure 11B, dark violet points and dark yellow points, respectively) and show significantly attenuated arousal responses to higher intensity UV light relative to *gl60j* (Figure 11B, dark violet points versus red points), *cry-null* (Figure 11B, dark violet points versus green points), and *gl60j-cry-null* (Figure 11B, dark violet points versus yellow points), underscoring the importance of Rh7 for UV light evoked arousal for this light intensity. This result is consistent with reports that Rh7 is a bistable broad range photopigment and is activated in the UV range (Ni et al., 2017; Sakai et al., 2017).

**FIGURE 12:**
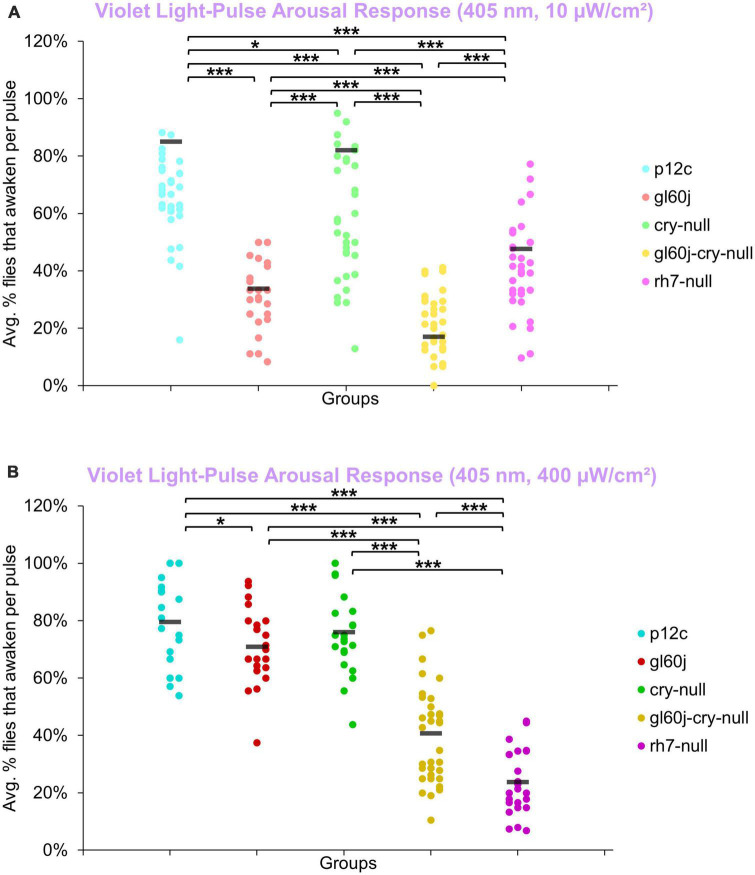
Violet light pulse arousal behavior is significantly attenuated in flies lacking Rh7 or external photoreceptors with low and high intensity light compared to the control *p12c*. Three 5 min pulses of violet light were applied to flies during subjective nighttime (ZT18, ZT19, ZT20) for 3 days after 12:12 h LD entrainment to measure the arousal response of *p12c* (blue), *gl60j* (red), *cry-null* (green), *gl60j-cry-null* (yellow), and *rh7-null* (violet) flies for **(A)** low (10 μW/cm^2^) and **(B)** high (400 μW/cm^2^) light intensity. Scatter plots are grouped by average% of flies that awaken across the 3 days of light-pulse arousal experiment and separated as pulse 1 (left points for each group), pulse 2 (middle points for each group), and pulse 3 (right points for each group). Black bars indicate total average% flies that awaken across the 3 days and three pulses of light. Pairwise comparison was analyzed using two-sample *t*-test with FDR adjustment. *p < 0.1 and ***p < 0.01.

An average of approximately 85% of control *p12c* flies are aroused from sleep in response to low (10 μW/cm^2^) intensity violet light (Figure 12A, light blue points). Low intensity violet light pulses evoke significantly lower arousal responses for all photoreceptor mutants compared to *p12c*, with *gl60j* and *gl60j-cry-null* showing the greatest attenuation of violet light evoked arousal (Figure 12A). Not surprisingly, *cry-null* alone shows the least attenuation of violet light evoked arousal as no redox state of CRY exhibits high absorption in the violet range of the spectra. Loss of Rh7 results in significant attenuation of the low intensity violet light response, but significantly less so relative to either *gl60j* or *gl60j-cry-null*. For high intensity violet light pulses, all photoreceptor mutants except *cry-null* flies show significantly attenuated arousal responses (Figure 12B), suggesting that the lack of CRY activation by violet light may mediate spectral differentiation for short-wavelength light arousal responses. Arousal of *rh7-null* flies is most significantly attenuated in response to high intensity violet light pulses (Figure 12B, dark violet points).

**FIGURE 13:**
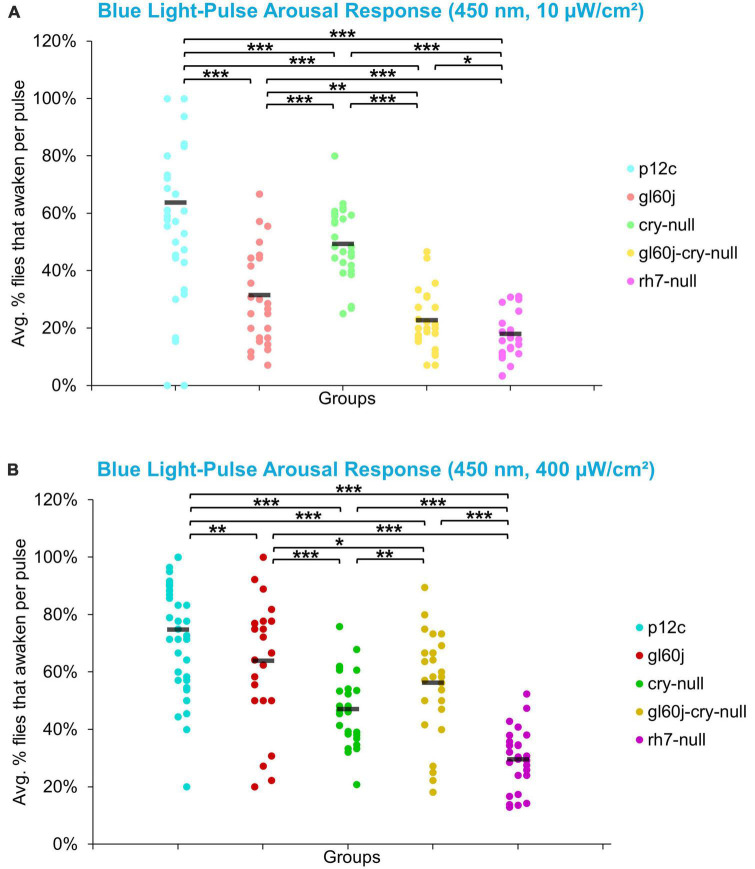
Low and high intensity blue light pulse arousal behavior is significantly attenuated in all photoreceptor mutants compared to the control *p12c*. Three 5 min pulses of blue light were applied to flies during subjective nighttime (ZT18, ZT19, ZT20) for 3 days after 12:12 h LD entrainment to measure the arousal response of *p12c* (blue), *gl60j* (red), *cry-null* (green), *gl60j-cry-null* (yellow), and *rh7-null* (violet) flies for **(A)** low (10 μW/cm^2^) and **(B)** high (400 μW/cm^2^) light intensity. Scatter plots are grouped by average% of flies that awaken across the 3 days of light-pulse arousal experiment and separated as pulse 1 (left points for each group), pulse 2 (middle points for each group), and pulse 3 (right points for each group). Black bars indicate total average% flies that awaken across the 3 days and three pulses of light. Pairwise comparison was analyzed using two-sample *t*-test with FDR adjustment. *p < 0.1, **p < 0.05, ***p < 0.01.

On average, approximately 65% of *p12c* control flies are aroused in response to low intensity blue light while approximately 75% are aroused in response to high intensity blue light (Figures 13A, B). All photoreceptor mutants exhibit significantly attenuated arousal responses compared to *p12c* control flies for both low and high intensity blue light (Figures 13A, B). The trends for the degree of attenuation of blue light evoked arousal responses are very similar to those measured for l-LNv blue light evoked electrophysiological action potential firing (Figure 3). Both low and high intensity blue light evoked arousal responses show *rh7-null* flies have the most attenuated response (Figure 13A, light violet points; Figure 13B, dark violet points). Interestingly, compared to *gl60j* and *gl60j-cry-null* flies, *cry-null* flies exhibit significantly less arousal response attenuation to low intensity blue light pulses (Figure 13A, light green points) but significantly greater attenuation responses to high intensity blue light pulses (Figure 13B, dark green points) which may reflect higher threshold for CRY blue light activation relative to the blue light activation threshold for opsins.

**FIGURE 14:**
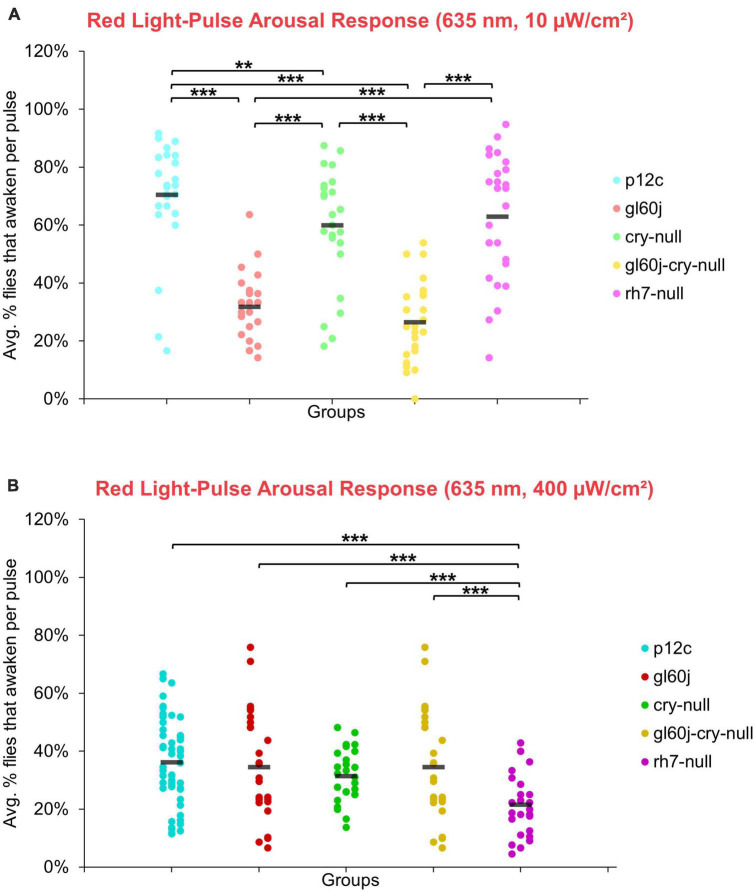
Red light pulse arousal behavior is significantly attenuated in flies lacking external photoreceptors at low and high light intensity compared to the control *p12c*. Three 5 min pulses of red light were applied to flies during subjective nighttime (ZT18, ZT19, ZT20) for 3 days after 12:12 h LD entrainment to measure the arousal response of *p12c* (blue), *gl60j* (red), *cry-null* (green), *gl60j-cry-null* (yellow), and *rh7-null* (violet) flies for **(A)** low (10 μW/cm^2^) and **(B)** high (400 μW/cm^2^) light intensity. Scatter plots are grouped by average% of flies that awaken across the 3 days of light-pulse arousal experiment and separated as pulse 1 (left points for each group), pulse 2 (middle points for each group), and pulse 3 (right points for each group). Black bars indicate total average% flies that awaken across the 3 days and three pulses of light. Pairwise comparison was analyzed using two-sample *t*-test with FDR adjustment. **p < 0.05 and ***p < 0.01.

On average, approximately 70% of *p12c* control flies are aroused in response to low intensity red light while less than 40% are aroused in response to high intensity red light (Figures 14A, B). Significantly fewer *gl60j*, *cry-null*, and *gl60j-cry-null* flies are aroused in response to low intensity red light relative to control (Figure 14A, light blue points vs. light red points, light green points, and light yellow points, respectively). Compared to *p12c* control flies, *rh7-null* flies do not significantly differ for low intensity red light pulse arousal responsiveness (Figures 14A, light blue points vs. light violet points). As the low intensity red light responses compared between *cry-null* and *gl60j-cry-null* flies do not significantly differ, external opsin-based photoreceptors appear to be the primary mediators of low intensity red light pulse arousal. High intensity red light evoked arousal response measurements further supports this (Figure 14B), as only *gl60j* (dark red points) and *gl60j-cry-null* (dark yellow points) flies are significantly less responsive compared to *p12c* controls (dark blue points).

**FIGURE 15:**
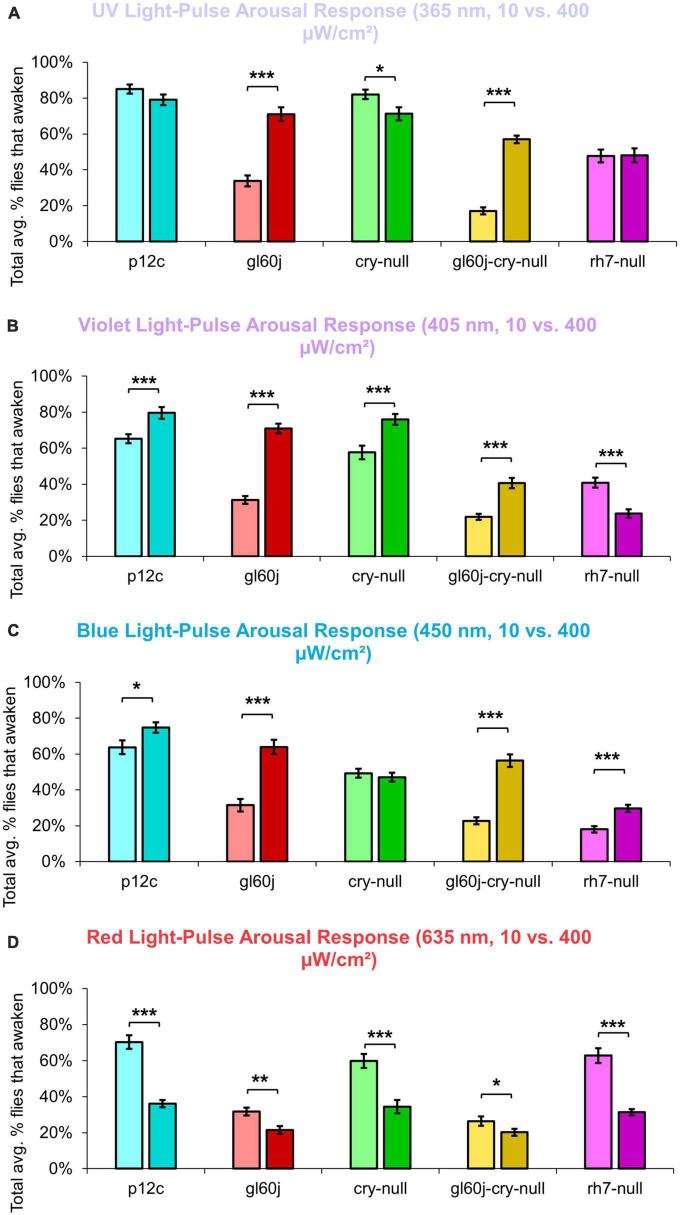
Pairwise summary comparison of light-pulse arousal between low and high intensity light. Light intensity comparison of total average% arousal response across 3 days and 3 pulses of light for *p12c* (lighter blue column, left, 10 μW/cm^2^; darker blue column, right, 400 μW/cm^2^), *gl60j* (light red, left, 10 μW/cm^2^; dark red column, right, 400 μW/cm^2^), *cry-null* (light green, left, 10 μW/cm^2^; dark green column, right, 400 μW/cm^2^), *gl60j-cry-null* (light yellow, left, 10 μW/cm^2^; dark yellow column, right, 400 μW/cm^2^), and *rh7-null* (light violet, left, 10 μW/cm^2^; dark violet column, right, 400 μW/cm^2^) flies for **(A)** UV, **(B)** violet, **(C)** blue, and **(D)** red light stimulus. Pairwise comparison was analyzed using two-sample *t*-test. *p < 0.05, **p < 0.005, ***p < 0.001.

The overall trend indicates that intensity matched short wavelength light more effectively arouses flies from sleep than long wavelength light. Curiously, for red light, significantly fewer flies are aroused by the higher intensity condition (Figure 15), while for blue light, significantly more flies respond to the higher intensity condition, except *cry-null* flies, which are effectively aroused for both intensity conditions (Figure 15). Violet light pulses also showed a similar trend, with all photoreceptor groups except *rh7-null* flies having an increase in arousal responsiveness to high intensity compared to low intensity violet light, while *rh7-null* flies significantly respond less to the high intensity condition compared to the low light intensity condition (Figure 15). A summary table of behavioral arousal responses to light pulse stimuli for all genotypes can be found on Table 2. Taken altogether, these results provide strong evidence of a multifaceted photoreceptor convergence system that inputs mechanistically distinct different channels of photic information to arousal neurons that correspond to different spectral wavelengths and different intensity-dependent light activation thresholds. Removal of any one of these photoreceptor systems results in a significant loss of l-LNv photoexcitability or downstream behavioral arousal, with partial remaining functionality indicating robustness through redundancy.

**TABLE 2:** Summary of average% flies that awaken from light pulse arousal for each genotype against each color of light stimulus.

	Low intensity (10 μ W/cm^2^)				High intensity (400 μ W/cm^2^)			
	**UV**	**Violet**	**Blue**	**Red**	**UV**	**Violet**	**Blue**	**Red**
*p12c*	0.8503	0.6519	0.6375	0.7036	0.7905	0.7954	0.748	0.3611
*gl60j*	0.3379	0.3132	0.3147	0.317	0.7097	0.7091	0.6392	0.2145
*cry-null*	0.8199	0.5766	0.4928	0.5992	0.7119	0.7597	0.4706	0.3443
*gl60j-cry-null*	0.1705	0.2189	0.2268	0.2641	0.5701	0.4067	0.5631	0.2023
*rh7-null*	0.4767	0.4086	0.1794	0.6283	0.48	0.2372	0.2961	0.3132

Total average% flies that awaken from light pulse arousal responses of *p12c*, *gl60j*, *cry-null*, *gl60j-cry-null*, and *rh7-null* for UV, violet, blue, and red (365, 405, 450, and 635 nm, respectively) light stimulus. Left half of table represents the low intensity 10 μW/cm^2^ light pulses, and the right half of the table represents the high intensity 400 μW/cm^2^ light pulses. The green shading represents greater arousal responses, while the white shading represents smaller arousal responses.

## 4. Discussion

Insects use a variety of sensory modalities to navigate their environments, including image forming and non-image forming vision. Remarkably, multiple critical light-driven behaviors are mediated through the clock gene expressing neural circuit in flies. In addition to regulating circadian behavior, this circuit also contributes to light activated behavioral arousal and phototaxis/photoavoidance light choice behavior. The circuit localization of these functions suggest that these light evoked behaviors may be modulated by time of day. The integration of multiple photosensory inputs for behavioral responses to light suggests the importance of functional redundancy as well as higher level processing of complex light spectral and intensity features. Circadian photoentrainment appears to mediated through a combination of external rhodopsin photoreceptors in the eyes and HB eyelets, as well as deep-brain photopigments CRY and possibly Rh7 (Emery et al., 1998, 2000; Stanewsky et al., 1998; Helfrich-Förster et al., 2001, 2002; Malpel et al., 2002; Rieger et al., 2003; Klarsfeld, 2004; Veleri et al., 2007; Sheeba et al., 2008a; Schlichting et al., 2014, 2015, 2016; Saint-Charles et al., 2016; Ni et al., 2017; Senthilan et al., 2019). These mechanistically distinct photoreceptors detect differences in light intensity, spectral composition, and exposure time. While the role of CRY for circadian entrainment is widely accepted, Rh7’s role is less clear (Kistenpfennig et al., 2017; Ni et al., 2017). Ni et al. (2017) show small but significant effects of Rh7 on circadian entrainment. Kistenpfennig et al. (2017) also show small but significant circadian effects in *rh7-null* mutants, their abstract states “However, in blue light (470 nm), Rh7 (0) mutants needed significantly longer to synchronize than wild-type controls, suggesting that Rh7 is a blue light-sensitive photopigment with a minor contribution to circadian clock synchronization. In combination with mutants that lacked additionally cryptochrome-based and/or eye-based light input to the circadian clock, the absence of Rh7 provoked slightly stronger effects.” The rich multiplicity of photosensory inputs suggests that these different input channels work together in a coordinated fashion, and likely extract sensory cues to determine precise time of day information. Such a system would allow further tuning of the circadian clock to respond to complex light cues that vary according to time of day, weather and season, particularly in the morning (Majercak et al., 1999; Chen et al., 2006; Bachleitner et al., 2007; Boothroyd et al., 2007; Picot et al., 2007; Stoleru et al., 2007; Vanin et al., 2012; Guo et al., 2014; Green et al., 2015; Breda et al., 2020). Other sensory modalities, such as temperature sensing also provide further cues. Similarly, light choice behavior, expressed as phototaxis versus photoavoidance, also relies on the combination of external rhodopsin photoreceptors in the eyes and HB eyelets and the deep-brain neuronal photopigments CRY and Rh7 (Heisenberg and Buchner, 1977; Miller et al., 1981; Gao et al., 2008; Yamaguchi et al., 2010; Schlichting et al., 2016; Baik et al., 2017, 2018). Recent work shows that light choice behavior varies by time of day and that the LNvs are a point of convergence for multiple light input channels that modulate light choice behavior (Baik et al., 2019b). The LNvs, particularly the l-LNv also serve as light activated arousal neurons that are embedded within the circadian neural circuit (Parisky et al., 2008; Shang et al., 2008; Sheeba et al., 2008b; Chung et al., 2009; Kilman et al., 2009). Yet how LNv circadian/arousal neurons functionally integrate different photic inputs to behavioral light arousal responses remained incompletely understood. Based on this set of earlier findings, we were motivated to measure the relative input contributions to l-LNv light evoked electrical excitation and behavioral arousal.

Short wavelength light evoked electrical excitation of the l-LNvs is mediated through all three photoreceptor systems. Our previous work has shown that upon blue and UV exposure of l-LNvs, CRY-mediated phototransduction increases in membrane electrical activity via the potassium channel subunit hyperkinetic (HK), an NADH [Nicotinamide Adenine Dinucleotide (NAD) + Hydrogen (H)] binding redox-sensor (Weng et al., 2006; Pan et al., 2008; Fogle et al., 2015). Using light-evoked electrophysiological assays that measure light evoked increases in action potential FF in positive control *w;pdfGAL4-p12c;* + flies, we record robust increases in FF following 5 s exposures of UV, blue, and violet light stimuli. By comparison, significantly attenuated short wavelength light responses relative to control are recorded most of the mutant photoreceptor knockout flies that lack either all external opsin-based photoreceptors (*gl60j*), or CRY (*cry-null*), Rh7 (*rh7-null*), and the double mutant *gl60j-cry-null.* Blue light stimulation evokes highly sustained FF increases for > 20 s before returning to baseline in controls. Blue light evoked sustained FF rates are significantly attenuated in each of mutant knockouts, showing that all three photosystems are critical for blue light sustained action potential firing rates. Violet light also evokes sustained action potential FF increases in controls, persisting for > 10 s, which are significantly attenuated in the mutant knockouts of photopigments that code for violet-sensitive rhodopsin-based phototransduction inputs (*gl60j*, *rh7-null*, and *gl60j-cry-null*). These results are consistent with previous findings that indicate CRY and Rh7 as the predominant blue and violet light internal photosensors, respectively, and show that external opsin driven photoreceptors also contribute additive/converging effects for blue and violet light sensing by the l-LNv. UV light exposure evokes significant, but less robust sustained increases in FF (< 10 s) in controls, which more rapidly return to baseline FF after the cessation of light. Short wavelength light evoked firing rate increases in l-LNvs are all significantly attenuated in *cry-null*, *gl60j* and *rh7-null* mutants and double knockout *gl60j-cry-null* relative to control except for violet light and *cry-null* mutants, showing a remarkable convergence of light inputs. The results are consistent with the earlier finding that Rh7 is expressed in the LNv as shown by Ni et al. (2017). UV, violet and blue light evoked increases in l-LNvs are all significantly attenuated in double mutant knockout *gl60j-cry-null* flies that lack all eye structures and do not express CRY. This provides support for the most parsimonious interpretation that Rh7 is expressed and is functional in the l-LNvs. There remains a formal possibility that Rh7 is expressed in brain neurons other than the l-LNv that would have to provide very strong synaptic inputs to the l-LNv that are sufficient to mediate light evoked firing of the l-LNv−however, this scenario also requires that such hypothetical strongly synaptically connected neurons are not detectable either by anti-Rh7 immunocytochemistry as reported by Senthilan et al. (2019). Another interesting, understudied question is whether yet unknown inhibitory interactions occur between the different light input channels converging on the l-LNvs as shown by behavioral studies in Fogle et al. (2015). The composite photosensitivity of light input channels may be modulated by the presence or absence of other photic inputs. In future work, we would like to explore the possibility that there may be yet unknown inhibitory interactions occurring between the different light input channels converging on the l-LNvs.

In contrast to short wavelength light, red light evokes relatively weak but still measurable excitation in the l-LNvs. Furthermore, the short wavelength sustained light evoked responses recorded in the l-LNv are not observed for red light responses. Surprisingly, red photoexcitation of the l-LNvs is dually regulated by CRY and external photoreceptors as shown by the significant attenuation of red light evoked action potential firing in neurons double knockout *gl60j-cry-null* flies compared with controls. Earlier work also shows that red light evokes minimal FF changes during the red light pulse that are not as sustained as light evoked action potential post-stimulus probability of firing increases evoked by short wavelength light (Baik et al., 2019a; Au et al., 2022). Further, l-LNv red light excitability has been found to attenuate with treatment with an FAD functional inhibitor, Diphenyleneiodonium (DPI), with *cry-null* mutants, or with a partial loss-of-function CRY point mutant that disrupts FAD photoreduction (Baik et al., 2019a). In our most recent study, we transgenically expressed CRY1 from a nocturnal mosquito species, *Anopheles gambiae*, in a *cry-null Drosophila* background and found those l-LNvs exhibit an even greater electrophysiological sensitivity to red light (Au et al., 2022). Although spectral absorption analysis of CRY’s FAD at oxidized and anionic semiquinone reduced states exhibit peak sensitivity primarily around blue and UV wavelengths, red wavelength sensitivity could occur if CRY expresses a biologically active neutral semiquinone FADH^•^ state. Altogether, external rhodopsins and CRY dually contribute to the l-LNv red light excitability in the present study, with previous work also supporting red light-excitatory CRY as an input to l-LNvs based on higher reduced states of FAD cycles. Additional experiments are required to dissect the exact external photoreceptive elements that provide red light signaling to l-LNvs and how they might interact with CRY and Rh7. Rh1 and Rh6 are likely candidates as they exhibit partial red light sensitivity and are expressed in photoreceptor cells that either directly or indirectly input to l-LNvs (Salcedo et al., 1999; Muraro and Ceriani, 2015; Schlichting et al., 2016).

We show representative firing records for each genotype tested and for each of the four light spectra employed in this study (UV, violet, blue and red). Consistent with most earlier publications, for our recordings, we observe predominantly tonic action potential firing in l-LNv recordings (Holmes et al., 2007; Cao and Nitabach, 2008; Sheeba et al., 2008b,^2010^; McCarthy et al., 2011; Seluzicki et al., 2014; Flourakis and Allada, 2015; Flourakis et al., 2015; Fogle et al., 2015; Buhl et al., 2016, 2019; Baik et al., 2017, 2019a; Li et al., 2018; Smith et al., 2019; Au et al., 2022). Burst firing as the predominant firing mode in l-LNv has been reported by another group (Muraro and Ceriani, 2015; Fernandez-Chiappe et al., 2021). It remains unclear why different groups see different firing patterns in l-LNv recordings. The baseline firing rate of l-LNvs varies by time of day (Cao and Nitabach, 2008; Sheeba et al., 2008b). Based on anatomical location of the l-LNvs embedded within the circadian neural circuit, we considered the possibility that light evoked excitation of the l-LNvs is circadian regulated. However, our experiment is not formally set up to test this hypothesis. The data set does not cover all 24 h time points, the majority of recordings were performed during daytime on an approximately 12:12 LD cycle. Using this limited data set, we do not detect significant time-of-day basal firing rate differences. Our data at present does not indicate significant time-of-day effects for the l-LNv electrical light responses, but further testing is required. A previous study from another group testing this question more comprehensively shows differences in l-LNvs electrophysiological properties and their responses to light in daytime versus nighttime recordings (Buhl et al., 2016).

The l-LNvs contribute to different light regulated behaviors of the fly, including circadian behavior, light choice attraction/avoidance behavior and arousal behavior (Sheeba et al., 2008a; Yoshii et al., 2012; Fogle et al., 2015; Baik et al., 2017, 2018, Schlichting et al., 2016). CRY is the primary circadian photopigment in *Drosophila melanogaster* yet is not required to maintain light-dark entrainment, since the fly clock has been shown to directly entrain by inputs from rhodopsins in each of the external photoreceptor systems: compound eyes, HB eyelets, and ocelli (Rieger et al., 2003). Specifically, Rh1, Rh3, Rh4, and Rh6 mediate low-intensity light re-entrainment properties of the clock (Saint-Charles et al., 2016), while Rh5 mediates medium and high intensity light re-entrainment, though this may occur via a non-PLC phototransduction pathway (Szular et al., 2012; Ogueta et al., 2018). It is thought that Rh6 expressing photoreceptor cells in the eyes converge all inputs from the outer and inner receptor cells in order to mediate circadian entrainment (Schlichting et al., 2014; Ogueta et al., 2018; Alejevski et al., 2019), though the precise anatomical characterization to clock neurons from these photoreceptor cells remains elusive. However, even with the removal of CRY and NORPA, flies were observed to still respond and entrain to light, leading to the discovery of internally expressed rhodopsin 7, which was found to also contribute to small but significant changes in circadian light entrainment (Ni et al., 2017) and a “minor contribution to circadian clock synchronization” (Kistenpfennig et al., 2017).

Light choice attraction/avoidance behavior is mediated by multiple photic inputs from the eyes, CRY, and Rh7 (Zhao et al., 2003; Dolezelova et al., 2007; Yuan et al., 2007; Keene et al., 2011; Rakshit and Giebultowicz, 2013; Schlichting et al., 2016; Baik et al., 2017, 2018; Alonso San Alberto et al., 2022). Each of these photic input channels have distinct features based on light intensity, spectral composition, and light exposure time. Specifically, acute (minutes) high-intensity (400 μW/cm^2^) and low-intensity (10 μW/cm^2^) UV light attraction is primarily mediated by external rhodopsin photopigments while long-lasting (tens of seconds) high-intensity (400 μW/cm^2^) UV light avoidance is primarily mediated by internal CRY and Rh7 photopigments.

In our present study, we provide evidence of multiple photic input integration for light arousal behavior. UV light-pulses show a significant attenuation in the arousal response of all rhodopsin-based phototransduction mutants (*gl60j*, *rh7-null*, and *gl60j-cry-null*) relative to controls for both low and high intensity lighting conditions, but not in *cry-null* mutant flies. Thus, fly arousal to UV light pulses is apparently opsin-dependent and CRY-independent. Unsurprisingly, violet light-pulses indicate the violet light-sensitive rhodopsins in the eyes and Rh7 as functional violet photosensors for violet light evoked arousal behavior. CRY appears to have a minor contribution for low-intensity violet light evoked arousal responses. Similarly, blue light evoked arousal responses depend on CRY and all rhodopsin photopigments (external and Rh7) for both low and high intensity blue light evoked arousal responses. These results suggest that functional redundancy is achieved by neutral integration of all three channels of photic input for blue light evoked arousal responses, while Rh7 activation may provide gain modulation for UV light evoked arousal responses. The average% of flies that awaken from low-intensity red light pulses is significantly attenuated relative to control with both the single knockout mutants *gl60j* and *cry-null*, as well as the double knockout mutants *gl60j-cry-null*. This finding closely matches the electrophysiological results for l-LNv electrophysiological recordings made from double knockout *gl60j-cry-null* flies. Surprisingly, with high-intensity red light pulses, the overall average% of flies that awaken are lower for all groups, and only *rh7-null* flies show significantly attenuated red light evoked arousal responses. This was a surprising observation that we believe suggests two possibilities: (1) the arousal neural circuit may have a detection threshold for red light that our 400 μW/cm^2^ high-intensity red stimulus exceeds, and (2) Rh7 has the highest intensity detection threshold amongst the three photoreceptor systems, but still requires input from external rhodopsins and CRY to enable a proper red light pulse arousal response.

There is a strong relationship between circadian neuronal electrical activity and clock cycling (Nitabach et al., 2002, 2005; Nitabach, 2006). There are only a handful of publications that measure clock protein cycling at high temporal-spatial resolution (Shafer et al., 2002; Roberts et al., 2015; Nave et al., 2021), and only a subset of those show the effects of 12:12 h light:dark cycles on the clock (Shafer et al., 2002; Nave et al., 2021). For clock driven behaviors, cryptochrome (CRY) is the primary circadian photoreceptor and mediates clock disruption by constant light, while eye light input is redundant to CRY (Helfrich-Förster et al., 2001; Nave et al., 2021). PER and TIM oscillations are highly synchronous across all major circadian neuronal subgroups in unshifted light schedules for 11 days. PER entry into the nucleus precedes TIM by about 3 h late at night (Shafer et al., 2002; Nave et al., 2021). A total of 3 h light phase delays followed several days later by 3 h light phase advances significantly dampens PER oscillator synchrony and rhythmicity in most circadian neurons during and after exposure. LNv clock protein oscillations are the first to desynchronize and the last to resynchronize following such light shifts, while the dorsal neuron group-3 (DN3s) within the circadian circuit increase their within-group synchrony in response to phase delay/phase advance light shifts. *In vivo*, alternating light shifts transiently disrupt sleep stability, and learning and memory processes, temporally coinciding with circuit desynchrony. The role of light shifts and subsequent clock circuit desynchrony is yet to be explored for other light evoked behaviors including light choice behavior and light evoked arousal.

Insect photobehaviors are evoked by many parameters of light, including intensity, spectral composition, and exposure time. Circadian photoentrainment and light attraction/avoidance behaviors function through the integration of multiple photic inputs. We provide additional evidence of a functional integration between these multiple sensory systems that converge input in l-LNvs to mediate neuronal photoexcitation and behavioral light arousal. Understanding how such complex light-evoked behaviors may allow us to target specific photoreceptor systems for more effective behavioral manipulations, which would lead a promising direction toward novel insect vector-control strategies (Grant et al., 1970; Chandler et al., 1976; Doukas and Payne, 2007).

## Data availability statement

The original contributions presented in this study are included in the article/supplementary material, further inquiries can be directed to the corresponding author.

## Author contributions

DA and TH designed the research, wrote, reviewed, and edited the manuscript. DA, JL, SP, MD, AF, and TN performed the research and analyzed the data. All authors contributed to the article and approved the submitted version.
